# Highly Pathogenic Avian Influenza (HPAI) H5N1 virus in Finland in 2021–2023 – Genetic diversity of the viruses and infection kinetics in human dendritic cells

**DOI:** 10.1080/22221751.2024.2447618

**Published:** 2025-01-02

**Authors:** Eda Altan, Veera Avelin, Kirsi Aaltonen, Essi Korhonen, Larissa Laine, Erika Lindh, Ilkka Julkunen, Niina Tammiranta, Tiina Nokireki, Tuija Gadd, Laura Kakkola, Tarja Sironen, Pamela Österlund

**Affiliations:** a Microbiology Unit, Finnish Institute for Health and Welfare, Helsinki, Finland; b Department of Virology, University of Helsinki, Helsinki, Finland; c Department of Veterinary Biosciences, University of Helsinki, Helsinki, Finland; d Institute of Biomedicine, University of Turku, Turku, Finland; e Veterinary Virology, Finnish Food Authority, Helsinki, Finland; f Clinical Microbiology, Turku University Hospital, Turku, Finland

**Keywords:** H5N1, highly pathogenic avian influenza (HPAI), Finland, genetic variation, infectivity, human cells, viral kinetic, immune cells

## Abstract

Highly pathogenic avian influenza (HPAI) H5N1 is known for its virulence and zoonotic potential, infecting birds and mammals, thus raising public health concerns. Since 2021 its spread among birds has led to cross-species transmission causing epizootics among mammals, eventually impacting fur animal farms in Finland in 2023. To analyze the infectivity of the Finnish H5N1 isolates in human cells, representatives of diverse H5N1 isolates were selected based on the genetic differences, host animal species, and the year of occurrence. The infection kinetics of the selected H5N1 isolates from wild pheasant and fox, and fur animals blue fox and white mink were examined in human monocyte-derived dendritic cells (moDCs) with H5N1 human isolate as a control. Although the isolate from pheasant (a wild bird) showed weakly reduced replication and viral protein expression in human cells compared to mammalian isolates, no discernible differences in virus replication in moDCs was observed. This study revealed similar infectivity in human moDCs for all five H5N1 isolates, regardless of the observed genetic differences. While H5N1 human infections remain rare, the virus poses a risk for widespread epizootics in mammals such as fur animal farms and, more recently, dairy cattle.

## Introduction

The emergence of the highly pathogenic avian influenza (HPAI) H5N1 virus as a zoonotic pathogen first became prominent during an epizootic event in Hong Kong in 1997. Although human cases have remained relatively rare, H5N1 has demonstrated a capacity for rapid transmission among avian species, with occasional spillover to mammals [1]. Over the decades, the virus has undergone significant genetic evolution through re-assortment events, allowing it to adapt to new hosts and environments. This evolutionary trajectory has been marked by the global spread of different HPAI strains, eventually in 2016 the HPAI H5N8 and H5N6 shown first time in Finland in wild bird populations. After this, the emergence of HPAI H5N1 in Finland led to outbreaks in both wild and captive birds, as well as sporadic infections in wild carnivores in 2021 [2]. In June 2023, the HPAI H5N1 virus caused mass mortalities among wild black-headed gull populations in Finland, and rapidly spread to fur farms causing a massive and severe epidemic in fur animals, resulting in significant losses especially among farmed minks [2–4]. In Spain, this same incidence in fur farms occurred in October 2022 and further analyses of the viruses from minks verified genetic changes similar as seen in human type influenza A viruses suggesting that this species could serve as a potential mixing vessel for the interspecies transmission [5,6]. Although no human cases were detected in Finland, these epizootic events highlight the apparent threat posed by H5N1 viruses to infections in mammals, and the need for continuous surveillance of emerging avian influenza viruses, based on research on species-specificity of the virus strains.

In humans, influenza A viruses infect epithelial cells of respiratory tract, followed by spread to dendritic cells (DCs) and macrophages (Mɸs) which reside in the immediate proximity of the epithelium [7]. DCs and Mɸs are important immune system cells that capture, process, and present antigens to T cells to initiate adaptive immune responses [8]. These cell types are important in replication studies of influenza viruses of different origin, since they express receptors for both avian-adapted (α-2,3-linked sialic acids) and human-adapted (α-2,6-linked sialic acids) influenza A viruses [9–11]. HPAI H5N1 viruses can infect human *in vitro* differentiated monocyte-derived DCs (moDCs), resulting in rapid viral replication and efficient spread of infection as compared to human seasonal and low pathogenic avian influenza viruses [12]. H5N1 virus infection in moDCs leads to cell death, impairs antigen presentation, and dampens the adaptive immune response, thus facilitating immune evasion [8]. H5N1 infection in DCs triggers an intense inflammatory and antiviral response, even in dysregulated manner leading to hypercytokinemia, “cytokine storm” [13]. Furthermore, H5N1 can exploit the migratory capacity of DCs to spread from the primary infection site to other organs, contributing to the systemic nature of the disease [8].

In this study, we analyzed the genetic diversity of HPAI H5N1 virus strains isolated from wild birds and mammals in Finland since 2021. We investigated the viral replication kinetics of the four most genetically distinct H5N1 viruses in human moDCs to understand the factors that influence viral replication and pathogenesis in human immune cells. This data is essential for developing effective strategies to mitigate the impact of H5N1 outbreaks and improve public health responses to potential zoonotic transmissions.

## Materials and methods

### Viruses

Sample of fourteen H5 virus strains were selected to this study representing the circulated H5 viruses in Finland since 2016. An early clade 1. human-originating HPAI H5N1 isolate from 2004 was selected as a control virus (Erasmus MC, Rotterdam, Netherlands). Human and animal isolates of avian influenza viruses were cultivated in the allantoic cavity of 10- to 11-day-old embryonated chicken eggs at +36°C for 2 days. Hemagglutination (HA) titration was performed according to standard protocols utilizing 0,5% rooster and turkey red blood cells. The infective virus titre was determined in Madin-Darby Canine Kidney (MDCK, ATCC CCL34) cells using a plaque assay with Avicel microcrystalline cellulose (FMC BioPolymers), and in moDCs by flow cytometry as described previously [12]. The number of plaques or infected cells per dilution was counted to obtain the amount of infectious particles as plaque forming unites (PFU)/ml or focus forming units (FFU)/ml, respectively, with 10-fold dilutions of the viruses up to 10^−9^. All infections were done without added TPCK-Trypsin.

### Next generation sequencing of H5N1 isolates

Viral genomic RNA of the 14 H5 isolated from embryonic eggs was extracted with the RNeasy mini kit (Qiagen). H5N1 genome segments were amplified according to the method by Zhou et al [14]. WGS libraries of the amplicons were produced using the Rapid Barcoding Kit 96 (SQK-RBK110-96, Oxford Nanopore), samples were loaded into a R9.4.1 flow cell (FLO-MIN106D, Oxford Nanopore) and run on an Oxford Nanopore MinION sequencing device for up to 12 hours. Raw reads were trimmed for adapter and primers used for avian influenza virus (AIV) amplification and the trimmed reads were mapped to the reference genome with 75% minimum overlap identity in Geneious program. All genomic segments were aligned using MAFFT in the Geneious software to identify variations among the isolates.

### Phylogenetic, network and mutation analyses

To construct the phylogenetic tree representing the hemagglutinin (HA) genome from European H5N1 strains, genomes were retrieved from GISAID spanning the period between January 1, 2018, and February 15, 2024. Only complete H5N1 genomes were included in the analysis. Initially, 5192 HA genomes from Europe were downloaded, aligned with MAFFT Alignment in Geneious program and subsequently genomes were concatenated based on similarity. From this process, 995 genomes were identified as suitable candidates for tree construction. H5N1 virus HA sequences (*n* = 995) were analyzed using the IQ tree program. To determine the optimal tree topology, model selection was performed using ModelFinder, which employs the Bayesian Information Criterion (BIC) to identify the best-fit model for the data. The selected model, GTR + F + I + G4, incorporates parameters for general time-reversible (GTR) substitution, among-site rate heterogeneity (G), proportion of invariable sites (I), and a discrete gamma distribution with four rate categories (G4). This comprehensive model was deemed the most suitable for accurately representing the evolutionary dynamics of the HA sequences under investigation. Furthermore, bootstrap analysis with 1000 replicates was employed to assess the robustness of the inferred phylogenetic relationships.

To reveal the genetic exchanges and evolutionary events shaping the diversification and dissemination of H5N1 strains in Finland, we ran a network analyses of HA genomes which shows that viruses collected from wild birds and mammals are closely related within the 2023 epizootic. We conducted Fluxus network analyses utilizing the complete set of Finnish HA genome sequences spanning from beginning 2023 onwards, comprising a total of 145 genomes. The goal was to elucidate the intricate evolutionary relationships among HA gene segments within the Finnish context. To achieve this, a phylogenetic network was constructed employing the Median Joining (MJ) method, a powerful tool for inferring evolutionary networks. Implemented within NETWORK 10.2.0.0 software, the MJ method utilizes a maximum parsimony approach to reconstruct connections between sequences, aiming to uncover the most parsimonious evolutionary pathways [15]. By applying this methodology to the HA gene segments, we finally included to the network analysis all the HA sequences from 2023, incorporating the pheasant and fox genomes from 2021 used in the study, for a total of 147 genomes.

A mutation table was compiled utilizing the GISAID Fluserver closest reference genome, database, and insights from Suttie et al. [16], which comprehensively synthesized available data on molecular markers/mutations in AIV. This compilation drew from diverse sources, including the CDC H5N1 Genetic Changes Inventory, the WHO Working Group on Surveillance of Influenza Antiviral Susceptibility (WHO-AVWG), and publications elucidating AIV mutations and molecular markers influencing biological traits and associated risks.

### Virus infection experiments in moDCs

Four representative H5N1 strains were chosen for infection experiments based on the genetic diversity, host species, and the year of detection, to cover outbreak events and different host species (Table 1). Human-originating H5N1 isolate from 2004, shown previously to replicate in human moDCs [12], was used as a control virus. Monocytes were isolated from buffy coat products obtained from anonymous blood donors from the Finnish Red Cross Blood Transfusion Service (permission no 47/2023, approved by the Finnish Red Cross Blood Service Institutional review board by which the need for informed consent was waived). The demographic status of anonymous blood donors (e.g. age, gender, infections or vaccination history) is not available. Human peripheral blood mononuclear cells were isolated through density gradient centrifugation over Ficoll-Paque gradient followed by Percoll gradient (Amersham Biosciences). T and B cells were depleted using magnetic beads (Dynal) to obtain a pure monocyte fraction. For the differentiation of moDC a previously described protocol was followed [12].

**Table 1.** Quantification of stock viruses used in study of Finnish animal-originating avian influenza H5N1 virus isolates from 2021–2023 with human-originating H5N1 from 2004.

Sample ID	Name	GISAID and GenBank IDs	HA titre		PFU/mL (MDCK)	FFU/mL (moDC)	vRNA copies per mL
			Rooster	Turkey			
Human/04	A/Vietnam/1194/2004	a	128	256	3.0E+07	1.3E+08	3.63E+09
White mink/23	A/white mink/Finland/UH-357-E1/2023	EPIISL18771244	256	256	2.4E+09	1.3E+08	3.26E+09
Blue fox/23	A/blue fox/Finland/UH-004-E1/2023	EPIISL18764855	128	256	1.3E+09	1.4E+08	3.43E+09
Fox/21	A/fox/Finland/2844_21VIR9619-1/2021	EPIISL15518268	256	1024	4.6E+09	2.2E+08	3.81E+09
Pheasant/21	A/pheasant/Finland/12309_21RS3535-4/2021	EPIISL15518264	256	1024	3.6E+09	2.8E+08	5.28E+09

^a^ AY651718, EF541402, AY651664, AY651610, AY651498, EF541466, EF541452, AY651552.

Each experiment was conducted using moDCs from four blood donors, with separate plate setups employed for cells, cellular RNA and protein samples. Experiments were replicated three times to ensure robustness and reliability of the results. Infection was carried out using different multiplicity of infection (MOI) values as indicated in the figures. MOI was calculated based on the FFU titres of the virus stocks determined in moDCs. Following infection, cells and supernatants were collected at 1-, 3-, 6-, and 24-hours post-infection.

### Flow cytometry

To assess the infectivity of studied H5N1 isolates in moDCs, cells from four blood donors were subjected to infection with six viral dilutions. At time points of 6 and 24 hours, the moDCs were harvested and fixed with 4% paraformaldehyde following the established antibody staining protocol with H5N1 glycoprotein specific antibodies followed by staining with secondary anti-rabbit-FITC antibodies (Caltag Laboratories) and respective FITC-conjugated rabbit isotype control was used [12]. The samples underwent analysis using a FACSCanto II (BD) instrument, operated with FACSDiva software. Briefly, live cells were gated from dead cell debris in FSC/SSC dot plot. In histogram plot from the gated live cells the background fluorescence was set with the sample stained with the isotype control antibodies and used for gating the uninfected cells. Fluorescence above the uninfected sample threshold was considered positive for viral glycoprotein staining.

### Western blotting

For protein analysis, the cells from different blood donors were pooled, and cell pellets were lysed using passive lysis buffer (Promega) supplemented with 1 mM Na_3_VO_4_. Total cellular proteins were separated on SDS-PAGE and transferred onto Hybond-P polyvinylidene difluoride membranes (Amersham Biosciences). Membranes were immunoblotted with specific antibodies. The rabbit antibodies against influenza A virus NP, M1 [12,17] and H5N1 glycoproteins were prepared as described previously and the specificity of these antibodies against H5N1 were shown previously [12]. Monoclonal rabbit antibody against GAPDH protein was used to control the equal loading of protein samples (Cell Signaling Technology, #2118). HRP-conjugated anti-rabbit IgG antibodies (Dako) were used as secondary antibodies. Proteins were visualized using Pierce™ ECL Western Blotting Substrate (Thermo Fisher) and imaged using iBright Imaging Systems (Thermo Fisher Scientific) with iBright Analysis software.

### qRT-PCR

Total cellular RNA was extracted using the RNEasy Mini kit together with RNase free DNase kit (Qiagen). The RNA was then reverse transcribed into cDNA using the TaqMan Reverse Transcriptase Kit (Applied Biosystems) with random hexamer primers. PCR was subsequently performed with an M1-specific primer and probe for influenza detection, alongside Gene Expression Assays for interferon-λ1 (IFN-λ1, Applied Biosystems; Hs00601677_g1). Based on prior studies, a minor modified version of the influenza M1 primers and probes designed by Ward et al. was selected [18]. These primers target the highly conserved M1 region, allowing detection of diverse influenza A viruses. The primers were validated using the map-to-primer method to ensure the detection of the strains used in this study. Data normalization was performed using 18S rRNA (Applied Biosystems). The relative gene expression compared to RNA from uninfected cells was calculated using the ΔΔCt method. The supernatant samples collected at 1- and 24-hours post-infection were analyzed with the M1 gene RT-qPCR along with a plasmid standard of known concentration to determine the viral particle quantities.

### Statistics

Statistical analyses were carried out with GraphPad Prism 10.2.3 (GraphPad Software). One-way ANOVA with Tukey’s multiple comparisons test was used to determine statistical significance.

## Results

### Genetic analysis and selection criteria of the H5N1 viruses

In this study, we selected a representative set of H5N1 isolates from our archival collection based on their genetic diversity. We aligned fourteen H5N1 Finnish isolates and whole European H5N1 isolates since 2020. When compared to H5N1 isolates submitted to GISAID from Europe since 2020, which exhibited minimum similarities of 82.9%, 87.2%, 84%, 97.7%, 79.9%, 61%, 93%, and 68% for PB2, PB1, PA, HA, NP, NA, MP, and NS segments respectively, four isolates we selected showed minimum sequence similarities of 91.1%, 97.9%, 91.9%, 98.3%, 85.1%, 97.7%, 98.7%, and 85.4% for the same segments (Supplementary Table 1). Notably, the HA gene, which plays a critical role in host receptor binding and antigenic properties, demonstrated a high degree of conservation, with our isolates showing 98.3 to 99.9% similarity compared to the 97.7 to 100% in European isolates. Our aim in this study was to assess the impact of H5N1 isolates circulating in Europe, as reflected in Finland, on moDCs. Based on the genetic diversity, host species, and the year of detection, H5N1 virus isolates from two distinct animal species (fox and pheasant) from the 2021 epizootic in Finland, along with two mammalian species (blue fox and white mink) from the 2023 fur farm epizootic, were selected for the study, with Human/04 H5N1 isolate as a control (Table 1) [12].

**Figure 1.**
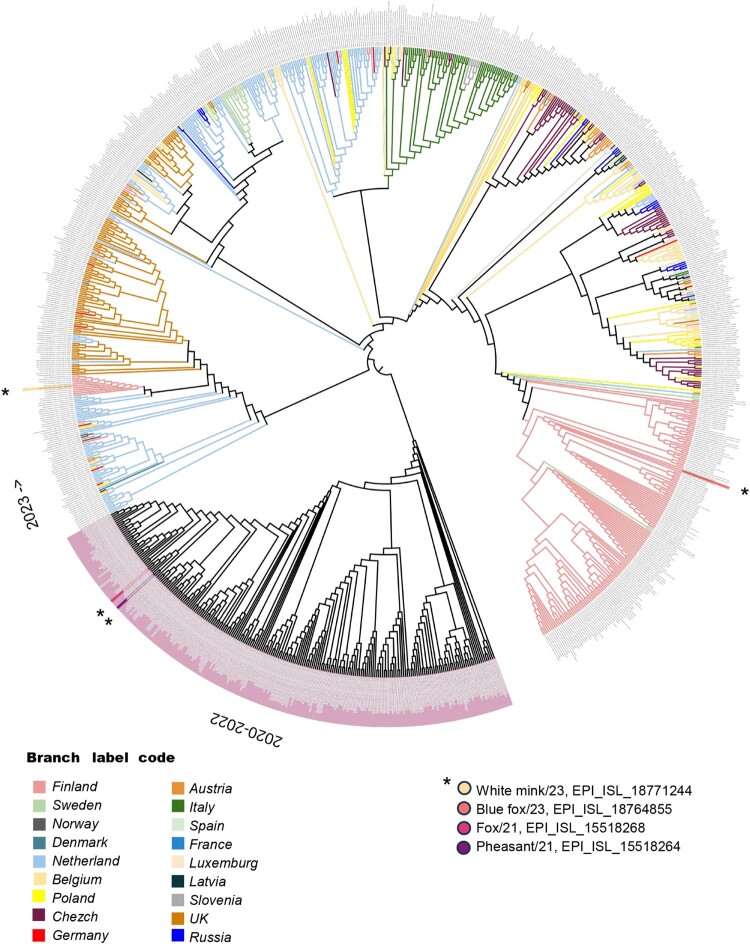
Phylogenetic tree of avian influenza H5N1 virus hemagglutinin (HA) genomes identified in Europe between the years 2020–2023. The phylogenetic relationship of highly pathogenic avian H5N1 virus HA sequences (n = 995) was analyzed using the IQ tree program. The tree was inferred by Bayesian analysis. H5 gene sequences from Europe between 2020 and 2022 are marked in purple and in 2023 labelled by country of origin. The Finnish mammalian and bird originating H5N1 virus strains selected for experimental analysis are marked in different colours and pointed with asterisk (*) in the figure.

Phylogenetic analysis revealed that the isolates clustered into three distinct branches (Figure 1). One branch primarily consisted of isolates from Finland, including our Blue fox/23 isolate, while the other branch with White mink/23 isolate was closely related to isolates from the Netherlands and Belgium in 2023. Additionally, the 2021 isolates from fox and pheasant clustered together within the 2020–2022 European branch (Figure 1).

**Figure 2.**
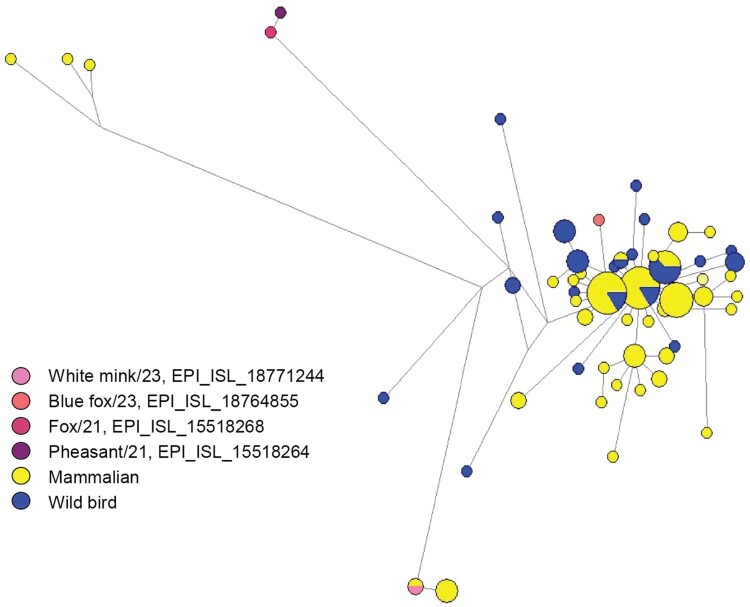
Phylogenetic network for the HA genomes of Finnish H5N1 viruses originating from mammals and wild birds from 2023 incorporated with the fox and pheasant isolates from 2021. The network is constructed employing the Median Joining (MJ) method, implemented within NETWORK 10.2.0.0 software. 147 HA genes originating from wild birds (*n* = 45 in blue), mammalian (*n* = 102, in yellow) were analyzed. The H5N1 virus strains selected for experimental analysis are marked in different colours as shown in the figure. The size of the circles represents the number of viruses of the same type, and the length of the branches refers to the nucleotide distance.

The network analysis of HA genomes suggests a close genetic relationship between H5N1 viruses collected from wild birds and mammals within the 2023 epizootic in Finland. This close alignment may imply cross-species transmission events or common sources of infection, highlighting the interconnected role of wild birds and mammals in the spread of H5N1 within this outbreak. The Blue fox/23 isolate exhibited a close genetic relationship with the predominant 2023 epidemic strains from Finland, while the White mink/23 isolate appeared more distantly related. Additionally, isolates from 2021 were positioned farther from the 2023 strains, indicating greater genetic divergence over time (Figure 2).

### Detected mutations in viral segments

Whole-genome analyses showed a diverse array of mutations distributed across eight genomic segments (Supplementary Table 1). While some mutations are known, based on the previous publications, as potentially significant, the others remain enigmatic, with their causal implications yet to be discovered.

HA protein is a critical surface glycoprotein facilitating cell attachment and membrane fusion. Based on the GISAID reference genome and identified significant mutations by Suttie et al [16], we observed 58 mutations and substitutions in the HA genome of our isolates (Supplementary Table 1). Notably, mutations such as S133A, S154N, S156A, V182N, and K218Q-S223R have been identified as potentially enhancing the binding of the virus to the human α2–6 receptor (Table 2). Additionally, we detected several side mutations in the HA genome, suggested to be associated with viral oligomerization interfaces, involvement in a T-cells epitope presented by MHC molecules, and binding to small ligands (Supplementary Table 1).

**Table 2.** Selected mutations potentially associated with zoonotic spillover events (according to previous publications) detected in the Finnish animal-originating avian influenza H5N1 virus isolates from 2021 to 2023. Human-originating isolate from 2004 as a control. Detailed description of all the mutations in Supplementary Table 1.

PB2	K389R	All	NP	V33I	2,3
	V598T	All		Y52N	2,3
	E627K	1,2,4		I109V	2,3
	G309D	All	NA	H155Y	2,3,4,5
PB1	S375N	All		T365I	2
	P598L	All		N366S	2,3,4,5
	D622G	All	M1	N30D	All
PA	S37A	All	NS1	P3S	All
	P190S	All		R41K	All
	H266R	All		K55E	All
	A343S	2,3		K66E	All
	S515T	All		D70G	2,3
HA^a^	S107R	All		L103F	All
	T108I	All		I106M	All
	S133A	2,3,4,5		C138F	All
	S154N	All		V149A	All
	T156A	2,3,4,5	1 Human/04 2 White mink/23 3 Blue fox/23 4 Fox/21 5 Pheasant/21		
	V182N	All			
	K218Q	All			
	S223R	2,3,4,5			
	K394E	All			

^a^ HA numbering based on H5 sequence.

Mutations in polymerase subunits are known to impact the replication potential of avian influenza viruses in mammalian cells, and especially E627K mutation has been implicated in enhancing polymerase activity, virus replication, pathogenicity, and mortality in mammals [19]. We identified three mammalian H5N1 isolates containing the E627K mutation in the polymerase subunit PB2 gene and two isolates (Blue fox/23 and Pheasant/21) lacking this mutation (Table 2). The T271A mutation in PB2, known to significantly enhance polymerase activity in human cells [20], was absent in all five analyzed isolates. However, each isolate possessed the K398R, V598T, L98V, and G309D mutations in the PB2 polymerase gene, which are associated with increased polymerase activity and replication efficiency in mammalian cell lines [20,21]. This combination of mutations makes it challenging to assess the individual contributions of each mutation to replication kinetics in moDCs replication. Though, an amino acid substitution, P534S, was identified solely in the PA genome of the Pheasant/21 isolate, setting it apart from the other isolates (Supplementary Table 1).

### Quantification of virus stocks

To reliably compare the infectivity of mammalian and avian origin H5N1 viruses in human cells, equal virus concentration is essential, and thus we titrated the stock viruses with three methods (Table 1). The HA titres were analyzed both with rooster and turkey blood cells, and we found that, although both cells should express the avian type receptors, the turkey red blood cells showed clearly higher HA titres for all the viruses. We also determined the titres of infectious viruses both in MDCK cells and in human moDCs. In MDCK cells the plaque forming units indicated that the infectivity of the Human/04 isolate was reduced by two logs compared to the animal-originating isolates. However, in human moDCs the infectious units analyzed as FFU titres, demonstrated that all virus stocks possess roughly equal infectivity in this cell model. Furthermore, the quantity of viral RNA with RT-qPCR verified the similar viral genome amounts in the stocks. These results emphasize the importance of selecting the titration method according to the downstream applications and thus the MOI for the infection experiments in moDCs was calculated based on the infectivity curves analyzed in moDCs.

### H5N1 infectivity in human moDCs

**Figure 3.**
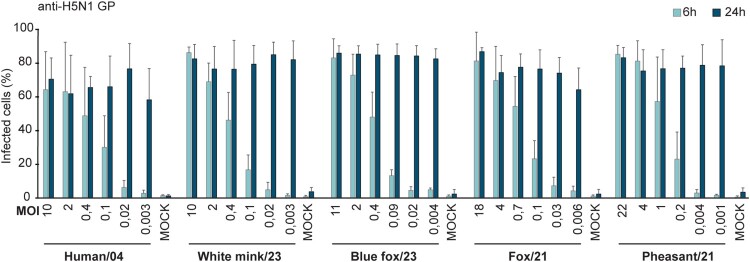
Infectivity curves of avian influenza H5N1 isolates in human monocyte-derived dendritic cells (moDCs). Cells from four different blood donors were infected with five H5N1 isolates with six one to five serially diluted virus doses starting from 1/75 dilution. Cells were fixed at 6 hours and 24 hours after infection and stained with anti-H5N1 glycoprotein (GP) specific antibodies followed by flow cytometric analysis to estimate the percentage of virus-infected cells. The mock sample, the uninfected cells, was used to separate the uninfected and infected cells. The results represent the mean values + standard deviations of the means from the cells of four different donors. The multiplicity of infection (MOI) was determined as 0.5 where 50% of the cells were infected, and the MOI for each dilution was calculated based on that.

To determine the infectivity of the selected four isolates in human moDCs, the cells were infected with serial virus dilutions for 6 hours (representing a single-cycle infection), and the proportion of infected cells was analyzed with flow cytometry. From the linear phase of the infectivity curve, the multiplicity of infection (MOI) was determined as 0.5 where 50% of the cells were infected, and the MOI for each dilution was calculated (Figure 3). Based on the infectivity curve, the single-cycle infectivity in human moDCs was similar across three virus isolates, resulting in roughly 50% of the cells infected with the third dilution of 1/2000 giving MOI 0.4 (Human/04, Blue fox/23 and White mink/23) whereas the earlier 2021 isolates gave slightly higher infectivity with this dilution resulting to MOI 0,7-1 (Fox/21 and Pheasant/21). Analysis of the multi-cycle infection (24 hours) revealed that the infectivity curves of the four animal-originating isolates resembled that of the Human/04 virus, which has been shown to spread extremely efficiently in moDCs [12], leading to the maximum infectivity even with a very low MOI (Figure 3).

### Virus replication in human moDCs

**Figure 4.**
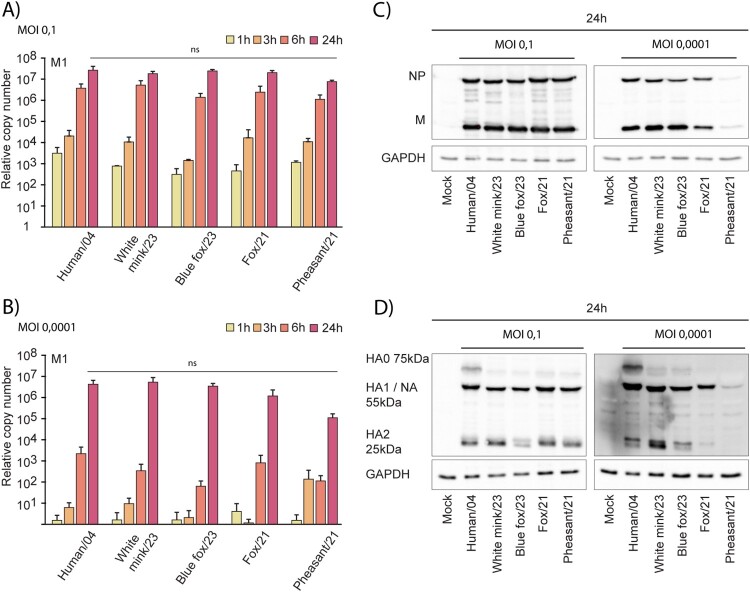
H5N1 virus replication in human monocyte-derived dendritic cells. moDCs from four blood donors were infected with five H5N1 isolates with MOI 0,1 and 0,0001. Cells were collected at 1-, 3-, 6-, and 24-hours post-infection, cells from different donors were pooled, and total cellular RNA was extracted. RNA samples were analyzed with influenza A M1 RNA-specific RT-qPCR. The results are presented as the expression of viral RNA relative to mock cell RNA in MOI 0,1 (A) and MOI 0,0001 (B) infected cells, with the average of three individual experiments with standard deviation. For the statistics One-way ANOVA with Tukey’s multiple comparisons test was used. Ns refers to not significant statistical differences between the samples. The expression of viral NP and M (C) and cleavage of the hemagglutinin (HA) protein (D) in cells collected at 24 hours post-infection. Cellular protein lysates from four donors were pooled and prepared for Western blotting and stained using virus protein specific antibodies. GAPDH staining was used as an internal loading control. A representative experiment out of three is shown. NA: neuraminidase that with the H5N1 glycoprotein antibodies cross react.

To assess the replication kinetics of the viruses, samples were collected from infected moDCs at 1-, 3-, 6-, and 24-hours post-infection for RNA and protein expression analyses. The replication kinetics of the H5N1 isolates, measured by RT-qPCR for viral RNA, was very similar in moDCs infected at MOI of 0.1, irrespective of the origin of the virus (Figure 4(A)). In the infection assays conducted at MOI of 0.0001, the Pheasant/21 isolate exhibited lower replication, approximately 2 logs less, over the 24-hour period (Figure 4(B)). Additionally, the expression levels of the NP and M1 proteins were reduced in the low MOI Pheasant/21 infection compared to the other isolates showing comparable levels of NP and M1 protein expression (Figure 4(C)).

Hemagglutinin protein of influenza viruses is synthesized initially as HA0, and the subsequent proteolytic cleavage into HA1 and HA2 subunits is imperative for the infectivity and endosomal fusion-mediated entry. Here, we investigated the cleavage of the HA protein of the four selected H5N1 isolates. An efficient cleavage of HA0 into HA1 and HA2 was observed in moDCs 24 hours post-infection with the higher MOI for all isolates (Figure 4(D)). However, again with the low MOI of 0.0001 the Pheasant/21 isolate showed low expression of HA and weakly detectable HA0 cleavage. Interestingly, during the infection with low MOI the cleavage of HA was clearly stronger in the fur animal-originating isolates from 2023 than in the wildlife isolates from 2021.

### Induction of antiviral IFN responses with H5N1 isolates

**Figure 5.**
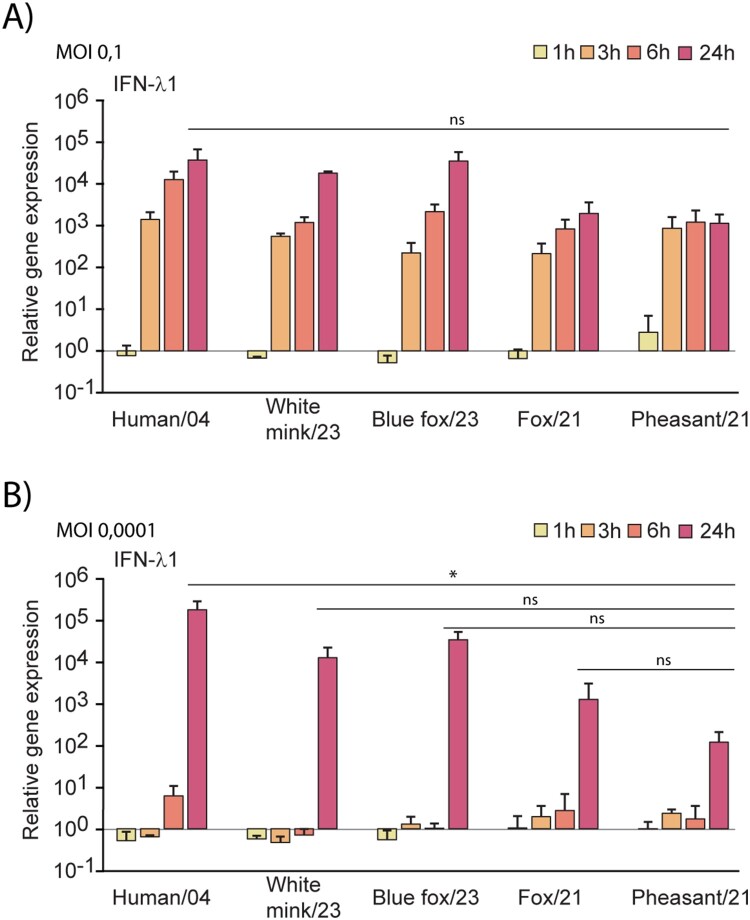
H5N1 virus-induced interferon lambda 1 (IFN-λ1) gene expression in human monocyte-derived dendritic cells. MoDCs were infected with five H5N1 isolates at MOI values 0.1 (A) and 0.0001 (B). Cells were collected at indicated time points after infection, pooled, and total cellular RNA was extracted. Relative IFN-λ1 gene expression was analyzed by RT-qPCR. The results are an average of three independent experiments and the data is presented as relative IFN-λ1 mRNA expression compared to uninfected cells. One-way ANOVA with Tukey’s multiple comparisons test was used for the statistics. *p* < 0.05 (*), not significant (ns).

To assess the virus-induced activation of antiviral innate immune responses, we quantified the expression of interferon lambda 1 (IFN-λ1) gene in infected human immune cells. Upon infection with MOI 0.1, a notable immunological response was observed within 3 hours post-infection (Figure 5(A)). However, when infected with low MOI 0.0001, increased IFN-λ1 mRNA expression was detected only at 24 hours post-infection (Figure 5(B)). In addition, the induction of IFN-λ1 mRNA was significantly weaker in response to infection with the Pheasant/21 isolate as compared to the Human/04 isolate (*p* < 0.05, 95% confidence interval (CI) 0.37 × 10^5^ to 2.97 × 10^5^, Supplementary Table 2).

### Productivity of virus infections in human moDCs

**Figure 6.**
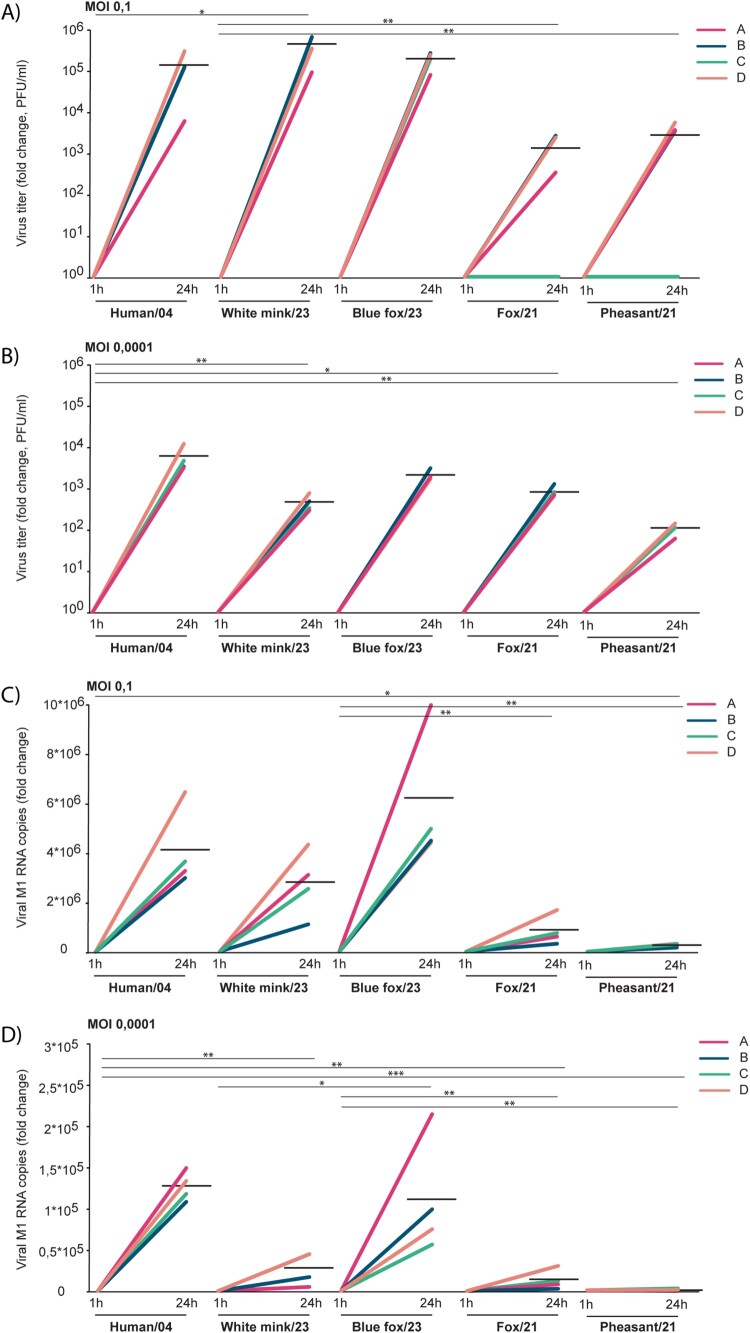
Productivity of H5N1 virus infections in human monocyte-derived dendritic cells. MoDCs obtained from 4 blood donors (marked with coloured A–D) were infected with H5N1 isolates at MOI 0.1 (A and C) and 0.0001 (B and D), and supernatant samples were collected at 1- and 24-hours post-infection. A and B. The infective viral titres produced from moDCs were determined by plaque assay in MDCK cells (logarithmic scale). C and D. Total RNA was extracted from supernatant and viral M1 RNA copies were quantified by RT-qPCR (linear scale). Virus titres and viral M1 RNA copy numbers were compared to the 1 h time point and are presented as fold change between 1- and 24-hours samples. Data of individual donors (A–D) are marked with indicated colours. The horizontal black lines represent the geometric means of the results from 4 blood donors. Statistics was run with One-way ANOVA with Tukey’s multiple comparisons test. *p* < 0.05 (*), *p* < 0.01 (**), *p* < 0.001 (***), not significant (ns).

Previously, we have shown that the Human/04 H5N1 virus infection in human moDCs results in production of infectious H5N1 progeny viruses [12]. Here we compared whether the currently circulating animal-originating H5N1 isolates can initiate similar productive infection in human moDCs. MoDCs from four donors were infected and the supernatant samples collected at 1 hour (contains input virus) and 24 hours post-infection were analyzed for the infectious progeny viruses with plaque assay (Figure 6(A,B)) and for the viral RNA with RT-qPCR (Figure 6(C,D), Supplementary Figure 1). As is shown in the Supplementary Figure 1 in this semi-attached moDC cell model as the input virus cannot be washed away the 1-hour samples contain still input viruses that have not entered the cells (except in Figure 6(B) where 1 hour sample with MOI 0.0001 PFU titres were all 0), and the productivity is thus analyzed as fold induction between the time points of 1 and 24 hours. The results clearly show that, like Human/04 virus, also White mink/23 and Blue fox/23 infections result in infectious progeny virus production in human moDCs, whereas infection with the Pheasant/21 isolate results in significantly less progeny virions (MOI 0,0001 Human/04 vs. Pheasant/21, *p* < 0.01, 95% CI 1.58 × 10^3^–9.92 × 10^3^, Figure 6, Supplementary Table 2). Based on plaque assay, the amount of live progeny virions seems to increase between 1 hour and 24 hours in cells infected with Pheasant/21 virus, however, the amount of viral RNA determined with RT-qPCR hardly exceeds the input level even at 24 hours after infection. These results verify that the mammalian-originating H5N1 viruses are able to productively replicate in human moDCs.

## Discussion

Highly pathogenic avian influenza (HPAI) H5N1 has caused devastating epizootic in wild and domestic bird populations worldwide since 2020. In July 2023, the virus made a concerning leap to Finnish fur farms [3]. The epizootic escalated dramatically and authorities reported HPAI H5N1 virus cases altogether in 71 fur farms leading to culling of ca 500,000 fur animals [4]. As also elsewhere in Europe, tens of thousands of wild birds died during the reported 36 outbreaks in Finland in 2023. H5N1 virus has infected also other wild animals and pets, for example [22]. The global H5N1 situation further escalated in March 2024 with H5N1 virus detected in US dairy cattle farms. Now the HPAI H5N1 virus has spread at least to 49 US states and 58 human cases have been reported [23]. This evolving threat underscores the critical need for vigilant monitoring and robust response strategies to mitigate the impact of the virus on both animal and human health.

In this study, we conducted a whole genome analysis of Finnish HPAI H5N1 isolates and other European HPAI H5N1 isolates. Fourteen Finnish H5N1 isolates, minimum sequence similarities across different genome segments, were aligned and compared to H5N1 isolates submitted to GISAID from Europe since 2020. The HA gene, which plays a critical role in host receptor binding and antigenic properties, demonstrated a high degree of conservation, with Finnish isolates showing 98.3–99.9% similarity compared to the 97.7–100% in European isolates. The whole genome analysis revealed a spectrum of mutations across all eight viral genomic segments. Among these we identified a mixture of mutations with potential biological effect on the virus. Notably, based on previous publications, certain mutations bear a significant relevance, potentially influencing critical biological processes in virus replication and virus-cell interactions. Conversely, other mutations remain enigmatic, lacking elucidation of their functional consequences. Our findings underscore the intricate landscape of genetic alterations within H5N1 virus genomic segments, highlighting both the known and unexplored drivers of genetic variation. Further investigation into the functional consequences of these mutations will enhance understanding of their impact on cellular physiology, disease etiology and pathogenesis.

Isolates used in the study clustered in three different branches in the phylogenetic tree suggesting potential differences in the evolutionary paths of these viruses. Although our study shows similar infectivity and replication results between these isolates except one of them, it does not include functional analyses to directly assess biological differences such as virulence or host adaptation. While no experimental data were collected to assess the biological differences between these clusters directly, it is possible that the clustering may reflect underlying genetic divergence that could influence factors such as host adaptation or transmission dynamics. Further investigation would be required to explore these possibilities in detail.

We further investigated the ability of HPAI H5N1 of different origin to replicate and induce antiviral interferon response in human cells using moDCs as a model for primary human immune cells. Four genetically distinct H5N1 isolates collected from different years and host species, including mammals and birds, were selected to investigate potential differences in viral replication and host innate immune responses. It is well acknowledged that human airway epithelial cells, macrophages and DCs are susceptible to HPAI H5N1 virus infection [7,9,10]. Study by Matthaei and colleagues shows that human-originating H5N1 viruses as well as seasonal virus are more prone to propagative infection in different type of human airway epithelial cells as the avian-originating H5N1 viruses [24]. They also suggest that H5N1 viruses need to be adapted to circumvent the strong IFN responses in human host cells. This is apparently mediated by NS1 known as a strong IFN antagonist of influenza A virus. Indeed, a previous study shows that switching a human or avian type NS1 protein into a recombinant influenza A virus leads to a different modulation of innate immunity in human DCs or epithelial cell models [25]. We have also previously shown that the human-originating H5N1 isolate from year 2004 replicates efficiently in human moDCs [12], and thus here we compared this virus isolate with both mammalian and avian-originating isolates from years 2021–2023 to reveal in what extent the mammalian-originating viruses might have acquired the adaptation towards human host. The findings of this study revealed that all five analyzed H5N1 virus isolates were able to infect, replicate and produce infectious viruses in human primary immune cells. In addition, there were no major differences observed among the five genetically different H5N1 isolates in high MOI in terms of their effect on the moDCs, however in low MOI the Pheasant/21 virus replicated less compared to the other isolates. Mutations in the H5N1 genomes are a critical area of research due to their potential impact on virulence, transmission, and antigenicity [16,26]. In this study, we found only one unique amino acid substitution in Pheasant/21 which is P534S in the PA genome, all other mutations in this isolate were common at least one of the other study isolates. The effect of P534S mutation on H5N1 is not yet known and, while we aim to avoid speculation regarding this mutation, we believe further comprehensive studies would be valuable on this mutation. Understanding the genetic variations, especially within HA, is essential for monitoring HPAI H5N1 evolution and designing strategies for surveillance, control, and prevention [27,28]. Even though the genetic analysis revealed several mutations between mammalian and avian isolates, the identified differences are not known to impact the viral replication in moDCs. While previous studies have linked some mutations to potential for mammalian infection, increased virulence, and drug resistance [16], our findings suggest none of the detected mutations in HA or polymerase subunits significantly affected viral replication kinetics in human moDCs.

Cells lining the physiological barrier surfaces, such as lung and intestinal epithelial cells, are particularly active at producing type III IFN-λs [29]. Consequently, respiratory epithelial cells preferentially produce IFN-λs in response to influenza A virus infections [29]. We have previously shown that H5N1 virus can induce IFN-λ1 in moDCs within the first three hours of infection with high MOI, and within 24 hours with low MOI [12]. Here, we obtained similar results with the virus isolates from 2023 epizootic, regardless of the origin of the virus isolates. The somewhat weaker IFN-λ1 response to infection with Pheasant/21 isolate is most probably due to the observed lower replication rate of the isolate as compared to other H5N1 isolates.

In conclusion, this study provides an analysis of the genetic diversity, replication kinetics, and host immune response of human, mammalian and bird originating HPAI H5N1 viruses in human moDCs. The findings highlight the significant genetic variability among H5N1 isolates, however, all of them were able, with only minor differences in their capacities, to infect and replicate in human moDCs.

## Supplementary Material

Supplementary Figure 1.pdf

Supplementary Table 2 Statistics.docx

Altan Supplementary Table.xlsx
