# Supplementary material for: Highly Pathogenic Avian Influenza (HPAI) H5N1 virus in Finland in 2021–2023 – Genetic diversity of the viruses and infection kinetics in human dendritic cells
Source: Emerg Microbes Infect. 2025 Jan 2;14(1):2447618. doi: 10.1080/22221751.2024.2447618 (PMC11727053; doi:10.1080/22221751.2024.2447618)
Supplement: Supplementary Figure 1.pdf [file TEMI_A_2447618_SM5577.pdf]

Supplementary Figure 1.

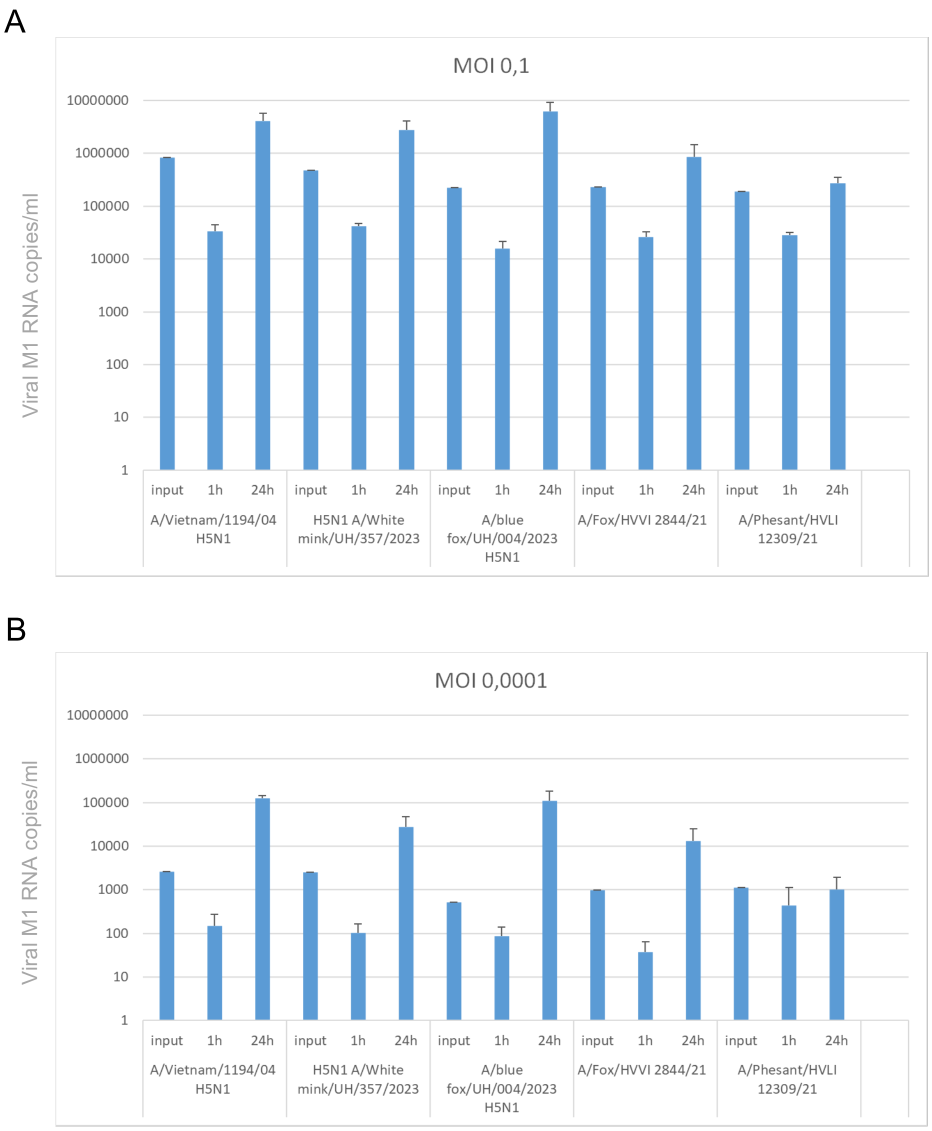

Supplementary Figure 1. Quantitation of input and output viral M1 RNA copies in supernatant samples collected at the beginning of the infection (input), 1 hour and 24 hours after infection of human moDCs. MoDCs obtained from 4 blood donors were infected with H5N1 isolates at MOI 0,1 (A) and 0,0001 (B), and supernatant samples were collected for viral RNA quantitation. Total RNA was extracted from supernatant and viral M1 RNA copies were quantified by RT-qPCR. Results are shown as means with standard deviations.
