# Supplementary material for: Highly Pathogenic Avian Influenza (HPAI) H5N1 virus in Finland in 2021–2023 – Genetic diversity of the viruses and infection kinetics in human dendritic cells
Source: Emerg Microbes Infect. 2025 Jan 2;14(1):2447618. doi: 10.1080/22221751.2024.2447618 (PMC11727053; doi:10.1080/22221751.2024.2447618)
Supplement: Supplementary Table 2 Statistics.docx [file TEMI_A_2447618_SM5576.docx]

Supplementary Table 2.

Mean differences, 95% confidence intervals (CI) and P-values for statistical comparisons. Analyses done with GraphPad Prism 10.2.3 (GraphPad Software, San Diego, CA, USA).
